# 嵌合抗原受体T细胞治疗相关神经系统毒副反应管理中国专家共识（2022年版）

**DOI:** 10.3760/cma.j.issn.0253-2727.2022.02.002

**Published:** 2022-02

**Authors:** 中华医学会血液学分会白血病淋巴瘤学组 中国抗癌协会血液肿瘤专业委员会造血干细胞移植与细胞免疫治疗学组

嵌合抗原受体T细胞（CAR-T细胞）已经发展成为难治复发血液肿瘤的有效治疗手段。在其临床应用不断增加的同时，治疗相关毒副反应受到越来越广泛的重视。神经系统并发症是CAR-T细胞治疗过程中常见的毒副反应之一，发生率为21％～66％[1]–[3]。随着CAR-T细胞治疗的不断开展，国内外对于治疗相关神经系统毒副反应的诊断和处理有了更深入的了解，中华医学会血液学分会白血病淋巴瘤学组和中国抗癌协会血液肿瘤专业委员会造血干细胞移植与细胞免疫治疗学组组织相关专家编写此共识，旨在帮助临床医务工作者对CAR-T细胞治疗过程中神经系统毒副反应的发生发展有更系统的认识，从而做到有效预防和治疗，提高CAR-T细胞治疗的安全性。

一、定义及发生机制

2017年Neelapu团队首先提出“CAR-T细胞相关性脑病综合征（CRES）”的概念，是指在CAR-T细胞治疗过程中，部分患者出现的头痛、谵妄、震颤、精神状态改变、注意力下降、语言障碍等神经系统异常表现[4]–[8]。2019年美国移植和细胞治疗学会（ASTCT）进一步提出了“免疫效应细胞相关神经毒性综合征（ICANS）”的概念[9]，即包括CAR-T细胞在内的免疫治疗后，患者内源性或外源性T细胞和（或）其他免疫效应细胞激活或参与而引起的一系列神经系统异常的临床表现。ICANS的定义较CRES更为准确地反映了神经系统病变的病理生理学特征，在目前临床诊断和治疗中获得了更广泛的认可和应用。本文使用ICANS命名CAR-T细胞治疗相关神经系统毒副反应。

ICANS的发生机制尚未完全明确。目前认为与CAR-T细胞治疗过程中单核/巨噬细胞活化介导的大量炎症因子释放有关[10]，而内皮细胞激活导致血管通透性增加、血脑屏障的完整性被破坏，IL-6、IFN-γ和TNF-α等多种细胞因子选择性通过血脑屏障，进入中枢神经系统，促进了ICANS的发展[11]–[12]。在发生严重ICANS患者的尸检病理中发现脑组织水肿，血管周围间隙扩大、液体外渗，皮质和白质星形胶质细胞损伤, 小胶质细胞激活等，证实炎症和血脑屏障破坏参与了ICANS的发生发展过程[13]–[14]。

目前已上市的针对CD19、BCMA CAR-T细胞产品[15]–[19]治疗相关ICANS发生率为28％～87％。在两项套细胞淋巴瘤患者接受CAR-T细胞治疗的真实世界研究中，ICANS发生率均在60％左右[20]–[21]。最近研究报道供者来源CD19 CAR-T细胞治疗急性B淋巴细胞白血病异基因造血干细胞移植后复发患者的ICANS发生率为21％[22]。ICANS发生率差异较大，与患者原发病、疾病状态、CAR-T细胞类型和输注剂量等因素有关。目前认为CAR-T细胞治疗前肿瘤负荷高、既往存在中枢神经系统损伤或肿瘤累及、输注高剂量CAR-T细胞，以及CAR分子结构包含鼠源单链可变片段（scFv）、使用CD28共刺激分子等，与ICANS发生率高有关[23]–[24]。

二、临床表现

ICANS临床表现多样，早期症状常表现为注意力减弱、语言障碍、书写能力减退等，可进一步发展为定向力障碍、情绪异常、失语、嗜睡、意识模糊和震颤等[3],[12],[25]，大多数患者ICANS临床症状呈可逆性[26]。少数患者可出现严重的临床症状，表现为癫痫发作、精神错乱、颅内压增高等。最严重的ICANS临床表现是急性脑水肿，患者可在数小时内从轻度的嗜睡进展为神志不清，进一步发展导致死亡[11],[13],[27]。

ICANS的症状和体征通常在CAR-T细胞输注后第3～6天出现，第7～8天达到高峰，后随着时间推移而逐渐改善，持续2～3周症状消失[10]。早期发生细胞因子释放综合征（CRS）的患者常合并严重ICANS[11]。大约10％的患者在CAR-T细胞治疗后第3～4周发生癫痫或谵妄等延迟性神经毒副反应。

鉴于ICANS临床表现复杂性和多样性，可运用量表对其进行分级[9]，指导疾病治疗。国际评分量表包括通用不良事件术语标准5.0（CTCAE5.0）、CAR-T细胞治疗相关毒性评分（CARTOX）和对CARTOX改良后的免疫效应细胞相关脑病评分量表（ICE）。临床工作中通常应用CARTOX-10和ICE评分系统，将ICANS分为四个等级。

三、辅助检查

在ICANS的诊断过程中，全面的神经系统检查非常重要，尤其是在疾病早期神经系统毒性症状表现不典型时。因此，对于既往有中枢神经系统疾病病史，或肿瘤累及中枢的患者，在CAR-T细胞输注前，需接受全面的神经系统评估，必要时请神经专科医师会诊。对于CAR-T细胞输注后所有患者，建议运用CARTOX-10或ICE评分量表，进行每天2次神经系统评估。当患者出现ICANS的临床表现时，应及时增加评估次数。除密切监测患者血常规、血生化、凝血功能、铁蛋白、细胞因子水平等指标，还需要进行以下几个方面检测。

1. 脑脊液检查：排除禁忌证后，可行腰椎穿刺和脑脊液（CSF）检查。发生ICANS时，常表现为颅内压升高，CSF检查可出现蛋白异常升高，偶尔可超过10 g/L，过高的CSF蛋白水平与预后不良相关[10]。CSF细胞计数通常轻度升高[21]，以淋巴细胞为主，部分患者CSF中通过流式细胞术可检测到CAR-T细胞[7]。大部分CAR-T细胞治疗后患者因骨髓抑制而并发血小板减少，腰椎穿刺术前应认真评估穿刺风险，必要时输注血小板后再行穿刺术。

2. 头颅CT/磁共振（MRI）：头颅CT有助于发现脑出血、脑梗死和脑水肿。头颅MRI表现多样，无特异性。T2WI或者FLAIR显示脑室旁、丘脑、延髓出现高信号，有时病变累及双侧，部分患者弥散加权成像（DWI）发现皮层高信号，提示出现细胞毒性水肿。小脑弥漫性水肿也有个例报道。大部分患者的影像学异常在症状缓解后可消失。及时的影像学检查有助于排除其他疾病，同时也有利于动态观察患者的神经系统毒副反应[10],[14],[28]。

3. 脑电图：常见的表现是弥漫性的慢波伴/不伴1-2Hz的三相波，部分患者出现纺锤波或癫痫波。大多数情况下，脑电图变化与临床症状发生具有一致性，脑电图异常与神经系统毒副反应的严重程度相关[20]，对高危患者，推荐脑电图动态监测[29]。

四、诊断及鉴别诊断

鉴于ICANS的临床表现多样，推荐多学科联合诊疗（MDT）模式进行临床诊疗。ICANS的诊断依据包括：

1. 患者在CAR-T细胞治疗后出现神经和（或）精神症状及相应体征。

2. 经CSF、头颅MRI、脑电图等检查，符合ICANS表现。

3. 排除其他神经系统疾病，主要包括：①颅内感染：可有精神症状，如谵妄、嗜睡等，常伴有发热等感染表现，推荐头颅增强CT或MRI评估颅内情况，可进行CSF常规、培养、病原学宏基因组二代测序等检查进一步明确病原学依据。②颅内出血：根据出血部位和出血量不同，临床表现有差异，多发生在患者骨髓抑制期严重血小板减少时；常起病较急，神经系统症状和体征在短时间内加重，伴有严重血小板减少时应警惕颅内出血发生可能，头颅CT平扫可进一步明确是否存在颅内出血。③原发病中枢累及：对于治疗前已有中枢累及或可能发生中枢累及的高危患者，应注意鉴别原发病的进展，必要时可进行CSF流式细胞术检测、头颅MRI等检查。

五、治疗

ICANS临床处理主要根据评分量表进行分层治疗（图1）。对已经出现神经系统症状的患者，应依据量表进行动态监测，根据患者病情变化，随时调整治疗策略。对于评分达到3～4级ICANS患者，建议转入重症监护病房，必要时予机械通气支持。在处置ICANS过程中，建议多学科联合治疗。

**图1:**
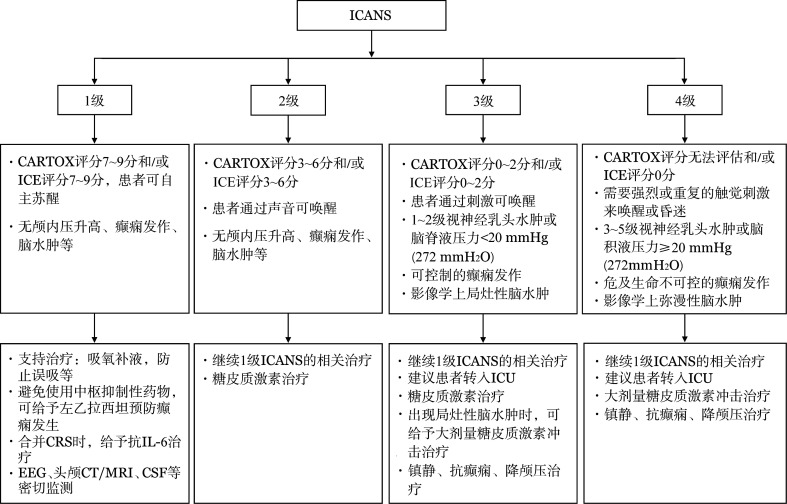
免疫效应细胞相关神经毒性综合征（ICANS）的分级与治疗流程 CRS：细胞因子释放综合征；IL-6：白细胞介素-6；EEG：脑电图；CSF：脑脊液；ICU：重症监护病房

1. 对症支持治疗：无论ICNAS等级如何，CAR-T细胞治疗后患者均应注意休息，可选择头部抬高体位（通常与床面呈30度），以增加静脉回流，防止误吸。吸氧补液，暂禁食禁饮，评估吞咽功能；加强营养支持，注意水、电解质平衡。积极控制体温，出现发热时可予物理降温措施和布洛芬等非甾体类药物，高热持续不退患者可使用冰毯辅助降温。

2. 糖皮质激素：在临床实践中，糖皮质激素常作为ICANS的一线治疗手段[3],[8]。糖皮质激素的使用是否会影响CAR-T细胞的疗效，目前尚无统一意见[30]–[32]。因此在密切监测患者病情变化的前提下，可遵循最低治疗剂量、最短治疗时间、快速减量的原则使用糖皮质激素，对于≥2级ICANS患者，可予地塞米松10 mg 每6 h 1次或甲泼尼龙1 mg/kg每12 h 1次方案治疗，直到ICANS等级降为1级后，开始减量，3 d内减停[33]–[34]。病情特别严重者可予甲泼尼龙每天1 g的方案冲击治疗2～3 d[34]。

3. 抗IL-6治疗：目前没有证据表明抗IL-6治疗可以降低ICANS的发生和延缓进展[10]，建议对所有等级的ICANS患者评估有无合并CRS。对于ICANS并发CRS的患者，可使用抗IL-6的治疗，建议IL-6受体拮抗剂托珠单抗（tocilizumab）每次8 mg/kg，单次最大剂量800 mg，必要时每8 h可以重复1次，最多使用4次。对于没有合并CRS的ICANS患者，不推荐使用托珠单抗治疗，因其有可能会导致IL-6浓度升高，诱发严重ICANS[35]–[36]。国外有研究报告使用IL-6抗体司妥昔单抗（siltuximab）每次11 mg/kg治疗，可直接结合IL-6，不会引起外周血或CSF中IL-6浓度升高。

4. 抗癫痫治疗：对于接受CAR-T细胞治疗的患者，尤其是既往有癫痫发作史、肿瘤累及中枢神经系统或头颅MRI、脑电图有异常的患者，在开始进行CAR-T细胞治疗时，可常规使用左乙拉西坦750 mg每12 h 1次口服预防癫痫的发生[12]。若出现癫痫发作，可选用经典的抗癫痫药物口服（卡马西平、丙戊酸钠、苯妥英纳等），或选用新型抗癫痫药物（拉莫三嗪、加巴喷丁、左乙拉西坦、奥卡西平和托吡酯等）。

对于惊厥性癫痫持续状态，初始治疗首选静脉地西泮10 mg，10～20 min内可酌情重复1次，或肌肉注射10 mg咪达唑仑。如果治疗效果不佳，可选择丙戊酸钠15～45 mg/kg（<6 mg·kg^−1^·h^−1^）静脉推注5 min后，续以1～2 mg·kg^−1^·h^−1^静脉泵注。若出现难治性癫痫持续状态，可选择静脉输注麻醉药物（丙泊酚、戊巴比妥、硫喷妥钠与咪达唑仑等）[37]。

对于非惊厥性癫痫持续状态，通常静脉给予地西泮10 mg，如治疗10 min后癫痫持续状态仍未终止，可相同剂量重复1次。如发作仍未控制，则静脉注射丙戊酸钠。如患者癫痫发作持续>60 min，建议开始应用麻醉药物，包括丙泊酚、戊巴比妥、硫喷妥钠与咪达唑仑等[38]。抗癫痫治疗过程中，需要密切关注患者呼吸循环系统，防止发生呼吸抑制。

5. 降颅内压治疗：对于存在1级或2级视乳头水肿，CSF压力<20 mmHg（272 mmH_2_O），且无脑水肿的患者：①床头抬高30度；②控制躁动，维持颅内压稳定；③尽可能缩短患者胸部物理护理（气管内吸痰、震动排痰、体位引流、叩背）时间（<30 min），以避免颅内压进一步增高。

对于存在3～5级视乳头水肿，伴影像学任何脑水肿征象，或CSF压力≥20 mmHg（272 mmH_2_O）的患者，应给予积极治疗[8],[39]，包括：①过度通气，PaCO_2_维持28～30 mmHg，不超过24 h。②脱水治疗：首先推荐使用20％甘露醇，初始剂量为0.25～1 g/kg；维持剂量每3～6 h 0.25～0.5 g/kg，同时每6 h监测电解质、内环境、容量状态及血浆渗透压等，治疗目标一般为维持血浆渗透压在300～320 mOsm/L，对于老年患者及肾功能容易受损的患者，治疗目标可为290～300 mOsm/L[40]。脱水治疗也可考虑使用高渗盐水（建议3％），推荐初始剂量3％高渗盐水250 ml，维持剂量每小时50～75 ml，同时每4 h 1次监测电解质，如果血清钠离子水平≥155 mmol/L，则停用高渗盐水。呋塞米联合甘露醇有助于提高降颅压疗效，可用于甘露醇单用疗效不佳患者。若肾功能不全，也可选用甘油果糖，但甘油果糖存在短时间颅压反弹现象，使用时需警惕。脱水治疗过程中，需严密监测患者生命体征，防止发生肾功能衰竭、电解质紊乱、血容量不足和低血压等，并需警惕脑水肿复发。③如果装有ommaya囊，可直接抽取CSF，直至CSF压力<20 mmHg（272 mmH_2_O）。④如上述治疗效果不佳，可请神经外科医师评估是否可行手术治疗。

六、预后

通常情况下ICANS预后良好，但少部分患者可进展为急性脑水肿等重症。在靶向CD19的CAR-T细胞治疗时，致命性神经系统毒副反应发生率约3％[11]，不容忽视。总之，目前ICANS的治疗方法有限，早诊断、早预防是管理ICANS的重要手段。
